# Structural racism theory, measurement, and methods: A scoping review

**DOI:** 10.3389/fpubh.2023.1069476

**Published:** 2023-02-16

**Authors:** Simone Wien, Andres L. Miller, Michael R. Kramer

**Affiliations:** Department of Epidemiology, Rollins School of Public Health, Emory University, Atlanta, GA, United States

**Keywords:** structural racism, population health, scoping review, theory, measurement, methods

## Abstract

**Introduction:**

Epidemiologic and public health interest in structural racism has grown dramatically, producing both increasingly sophisticated questions, methods, and findings, coupled with concerns of atheoretical and ahistorical approaches that often leave the actual production of health or disease ambiguous. This trajectory raises concerns as investigators adopt the term “structural racism” without engaging with theories and scholars with a long history in this area. This scoping review aims to build upon recent work by identifying current themes about the incorporation of structural racism into (social) epidemiologic research and practice with respect to theory, measurement, and practices and methods for trainees and public health researchers who are not already deeply grounded in this work.

**Methods:**

This review uses methodological framework and includes peer-review articles written in English published between January 2000–August 2022.

**Results:**

A search of Google Scholar, manual collection, and referenced lists identified a total of 235 articles; 138 met the inclusion criteria after duplicates were removed. Results were extracted by, and organized into, three broad sections: theory, construct measurement, and study practice and methods, with several themes summarized in each section.

**Discussion:**

This review concludes with a summary of recommendations derived from our scoping review and a call to action echoing previous literature to resist an uncritical and superficial adoption of “structural racism” without attention to already existing scholarship and recommendations put forth by experts in the field.

## 1. Introduction

Epidemiologic, public health, and quantitative population health's interest in “social determinants of health” (SDOH) has grown dramatically over 30 years, producing both increasingly sophisticated questions, methods, and findings, coupled with concerns of atheoretical and ahistorical approaches that often leave the actual production of health or disease ambiguous (1). Against this backdrop the intersection of inequities surfaced by the pandemic and racialized violent policing have elevated interest and calls for attention to a particular type of social determinant: structural racism. Documented as early as the nineteenth century by sociologist and U.S. “father” of social epidemiology, WEB DuBois in his work *The Philadelphia Negro* made the foundational contribution of characterizing how structural racism in the U.S. at the end of the nineteenth century produced racialized health trajectories for Black Americans (2–4). DuBois' insights also apply to historical harms to Indigenous communities for the past 600 years, and continue to be as salient in the twenty-first century for Black, Indigenous and other marginalized and racialized communities in the U.S. (5). Defined as “‘the totality of ways in which societies foster [racial] discrimination, *via* mutually reinforcing [inequitable] systems…that in turn reinforce discriminatory beliefs, values, and distribution of resources,' reflected in history, culture, and interconnected institutions,” structural racism is a sub-component of systemic racism, and is a fundamental cause (i.e., root cause) of health inequities (6, 7).

Measuring structural racism and considering opportunities for structural intervention is important for studies examining health inequities by racial stratification. While over the past 20 years (2000–2020) there has been a focus on mostly individual- and community-level social determinants of health, behind many of these determinants of health are structural determinants, including structural racism. This in turn can shape the maldistribution of social determinants of health (8). Said another way by Crear-Perry et al. (8).

*Individuals are unlikely to be able to control directly many of the upstream determinants of health: governance, policy, and cultural or societal norms and values that shape who has access to health-promoting resources and opportunities and who does not. Beginning from this vantage point allows an understanding of why social determinants are born from structural determinants and cannot be addressed separately. In other words, no matter how empowered, knowledgeable, or willing someone is to change their behavior, they may not be able to do so because of structural determinants of health inequities*.

Despite the increasingly sophisticated theoretical and conceptual advances, and perhaps in part because of the marginally increased funding for SDOH research over two decades, there is concern that “SDOH” has become so vague and diffuse as to lose explanatory and conceptual meaning (8–10). Reflection on this trajectory raises concerns that the same may be happening now for “structural racism,” as population health trainees enter the field and investigators at all stages with minimal experience on structural racism and health adopt phrases without engaging with theories and scholars with a long history in this area (10, 11). This practice of superficial engagement (even if unintended) and lack of accountability in research can result in “health equity tourism” (11–14). In the seminal article by Lett et al. health equity tourism is defined as “the practice of investigators—without prior experience or commitment to health equity research—parachuting into the field in response to timely and often temporary increases in public interest and resources…oftentimes, these scholars seek to ‘retrofit,' or adapt existing structures and research practices for health equity work, rather than build the necessary transformative infrastructure required for and sustainable health justice” (11). The consequences of health equity tourism are not innocuous: health equity “tourists” at best produce ineffectual work, and at worst reinforce harmful systems, structures, and beliefs, detract from important work done by scholars with long-standing commitments or expertise in health equity, and waste resources (11).

### 1.1. Purpose

The question that guided this review was “What are the contemporary themes for the epidemiologic study of structural racism with emphasis on theory, measurement, and methods?” This scoping review aims to identify current themes from extant literature about the incorporation of structural racism into (social) epidemiologic research and practice for trainees and public health researchers who are not already deeply grounded in this work. This paper synthesizes some key themes in the literature and highlights a few studies in-depth with the goal of providing guidance to trainees and epidemiologists interested in learning more. This review is divided into three sections. First, summarizing the seminal work by Adkins-Jackson et al. (12) and Hardeman et al. (13), we review conceptual approaches to structural racism that have developed over the past 20 years, including the importance of historical processes and the use of “actor-less” theoretical framing and measures. Second, we review how structural racism measurement has been operationalized, including considerations for data to create these variables. Lastly, we discuss practices and methods with respect to study design, collaboration, and statistics commonly used in structural racism health research. We conclude the review with a brief, non-exhaustive summary of best practices and a call to action put forth by previous scholarship for all population health scientists to engage in this work responsibly and equitably.

## 2. Methods

This scoping review uses the methodological framework developed by Arksey and O'Malley (15) (Table 1). Table 1 outlines our search strategy using Arksey and O'Malley's five-step framework: identifying the research question, identifying relevant studies, study selection, charting the data, and summarizing and reporting the results.

**Table 1:** Overview of scoping review.

**Step 1: Identify the research question(s)**	**What are the contemporary themes for the epidemiologic study of structural racism with emphasis on theory, measurement, and methods?**
Step 2: Identify relevant studies	1) Electronic databases: a Google Scholar search was conducted during February 2022 of English-language studies published between January 2000 and February 2022 using the following keywords: 1/1/2000–12/31/2019 • Structural framework health • Structural theory health • Structural measurement health • Racial capitalism health • Institutional racism framework health • Institutional racism theory health • Institutional racism measurement health • Meso determinants framework health • Meso determinants theory health • Meso determinants measurement health • Macro determinants framework health • Macro determinants theory health • Macro determinants measurement health • Social determinants racism framework health • Social determinants racism theory health • Social determinants racism measurement health 1/1/2020–2/27/2022 • Structural racism framework health • Structural racism theory health • Structural racism measurement health • Racial capitalism health
	The number of results for a given search term ranged from 17,000 to 2,410,000 articles; articles listed beyond approximately the 50th citation in Google Scholar were not reviewed
	2) Existing networks: these included articles shared by colleagues, professional society communications (e-mail, website), and Twitter by all authors (SW, AM, MK) from February to August 3) Citation engines: for select articles, CoCites was used to find other relevant articles that were frequently cited together 4) Reference lists: references of interest that were found from articles selected by any of the aforementioned search methods were included by all authors
Step 3: Study selection	Irrelevant and duplicate articles were identified by one reviewer (SW) at the abstract and title level. Articles whose main focus was not structural racism were excluded
Step 4: Extract the data	A data extraction form was developed to extract the following from each study if applicable
	Theory
	1) Discussion of or new conceptualizations of structural racism 2) Discussion of historical processes that contribute to structural racism 3) Explicit discussion of accountability in the conceptualization of structural racism, or who is harmed and/or benefits from structural racism
	Measurement
	4) What variables were used to operationalize structural racism, and if structural racism was a uni- or multi-dimensional measure; if the study was qualitative, what domains of structural racism were developed as a result of the study
	Practice and methods
	5) What method(s) were used to calculate the main measure of association, or if the study was qualitative, specify method(s) used
	Measurement, practice and methods
	6) If the study was a review of exposures or methods for structural racism and health
	All authors independently reviewed articles to extract the data. Any discrepancies with respect to the 6 topics were followed by a more in-depth reading of the article and discussed as a group until an agreement was reached
Step 5: Collating, summarizing, and reporting the results	All authors collectively reviewed data extraction summary for all included articles. Articles were grouped into three themes: theory, measurement, and practice and methods. Papers that were exemplar of these themes or included novel and/or interesting measures were included in a final table (Table 2)

Due to the interdisciplinary nature of structural racism studies (12), the use of several interchangeable terms to describe structural racism before 2020 (12), and our goal to provide an overview of relevant themes for structural racism and health, we made the following decisions: first, we chose to conduct a literature search of articles written in English using Google Scholar (as opposed to PubMed) in February 2022 in order to include articles from a variety of disciplines beyond biomedical research, as some articles are available in Google Scholar but not PubMed (16–31). Second, we chose two different sets of search terms for Google Scholar for pre- and post-2020, as several articles pre-2020 that discuss structural racism and its effects on health do not include the term “structural racism” (7, 9, 16–22, 32–58). Third, articles listed beyond approximately the 50th citation in Google Scholar were not reviewed. Search terms in Google Scholar yielded 17,000–2,410,000 articles, and are ranked by Google Scholar “…weighing the full text of each document, where it was published, who it was written by, as well as how often and how recently it has been cited in other scholarly literature” (59). We used this ranking to indicate an article's (provisional) relevancy in the literature. Lastly, given that key articles would be inevitably missed by this method, per Arksey and O'Malley's framework additional articles were added as a result of existing networks (colleagues, professional society messaging, Twitter), citation engines (CoCites) (60) and articles referenced in literature to identify key articles otherwise not generated from the Google Scholar search. These articles were collected from February 2022 to August 2022.

Articles were excluded if the main focus of the article was not structural racism (e.g., focused on structural determinants of health at large). Articles were read at the title and abstract level by one reviewer (SW), and duplicate articles or articles whose main focus was not structural racism were removed.

A data extraction form was developed to collect the following from the remaining 138 studies: (1) whether there was a discussion of or new conceptualizations of structural racism, (2) whether there was a discussion of historical processes that contribute to structural racism, (3) whether there was an explicit discussion of accountability in the conceptualization of structural racism, or who is harmed and/or benefits from structural racism, (4) what variables were used to operationalize structural racism, and if structural racism was operationalized as a uni- or multi-dimensional measure, (5) what method(s) were used to calculate the main measure of association, or if the study was qualitative, specify method(s) used; if the study was qualitative, what domains of structural racism were developed as a result of the study, and (6) if the study was a review of exposures or methods for structural racism and health. Questions 1–3 of the data extraction form correspond to articles that were focused on theory, question 4 corresponds to articles focused on measurement, question five corresponds to articles focused on practice and methods, and question six corresponds to both measurement and practice and methods.

All authors (SW, AM, MK) independently reviewed articles to extract the data. Any discrepancies with respect to the 6 topics were followed by a more in-depth reading of the article and discussed as a group until an agreement was reached. The summary of the articles exemplary of theoretical and methodological approaches to structural racism are presented below in tabular format (Table 2).

**Table 2:** Articles exemplary of theoretical and methodological approaches to structural racism in epidemiological research.

**References**	**Theory**	**Measurement**	**Practice and methods**
Adkins-Jackson et al. (61)	X	X	
Adkins-Jackson et al. (12)	X	X	X
Agénor et al. (62)		X	X
Alson et al. (63)	X	X	
Bailey et al. (6)	X		
Bailey et al. (64)	X		
Bonilla-Silva (21)	X		
Chambers et al. (65)		X	
Chambers et al. (66)		X	
Chambers (67)		X	X
Chantarat et al. (68)		X	X
Crear-Perry et al. (8)	X		
Dennis et al. (5)	X		
Dougherty et al. (69)		X	X
Feyman et al. (70)			X
Ford and Airhihenbuwa (71)	X		X
Gee and Ford (72)	X		
Gee and Hicken (73)	X		
Gee (55)			X
Gee et al. (40)	X		
Geronimus and Thompson (19)	X		
Geronimus (50)	X		
Graetz et al. (74)		X	X
Greene et al. (75)			X
Greer et al. (53)		X	
Groos et al. (76)		X	
Hamilton et al. (77)			X
Hardeman and Karbeah (78)	X		
Hardeman et al. (79)		X	
Hardeman et al. (80)	X		
Hardeman et al. (81)		X	
Hardeman et al. (13)	X	X	X
Hardeman et al. (82)	X		X
Hicken et al. (83)	X		
Homan et al. (84)	X	X	
Homan and Brown (85)		X	
Jahn (86)		X	
Jahn et al. (87)			X
Jones (51)	X		
Jones (20)	X		
Jones (37)	X		
Kramer et al. (32)		X	
Krieger (57)	X	X	
Krieger (41)	X		
Krieger (7)	X		
Krieger (88)	X	X	
Krieger (89)	X		
Krieger et al. (54)		X	X
Krieger et al. (33)			X
Krieger et al. (90)		X	
LaFave et al. (91)		X	X
Lett et al. (92)		X	
Lett et al. (11)			X
Lett et al. (93)			X
Lodge et al. (94)		X	X
Lodge et al. (95)		X	
Lukachko et al. (96)		X	
McClure et al. (97)	X		
Mendez et al. (58)		X	
Mesic et al. (98)		X	
Phelan and Link (99)	X		
Priest and Williams (100)			
Riley (101)	X		
Riley (102)	X		
Roach (103)	X		
Sabo et al. (104)			X
Sances and You (16)		X	
Sewell (105)	X	X	X
Sewell (106)	X	X	
Szanton et al. (107)	X		
Taylor (108)	X		
Thompson et al. (109)		X	X
Ture and Hamilton (22)	X		
Wallace et al. (110)		X	
Wallace et al. (111)			X
Walsemann et al. (112)		X	
White et al. (4)	X		
Williams and Mohammed (38)	X		
Williams and Mohammed (39)	X		
Williams and Sternthal (45)	X		
Williams (24)	X		
Williams et al. (46)	X		
Williams et al. (113)	X		
Williams and Collins (17)	X		

Given the guiding research question of identifying contemporary themes of structural racism in epidemiologic studies, there were no study criteria with respect to participants, interventions, or comparators for this scoping review. This scoping review was reported using the Preferred Reporting Items for Systematic reviews and Meta-Analyses extension for Scoping Reviews (PRISMA-ScR) Checklist (114).

## 3. Results

**Figure 1:**
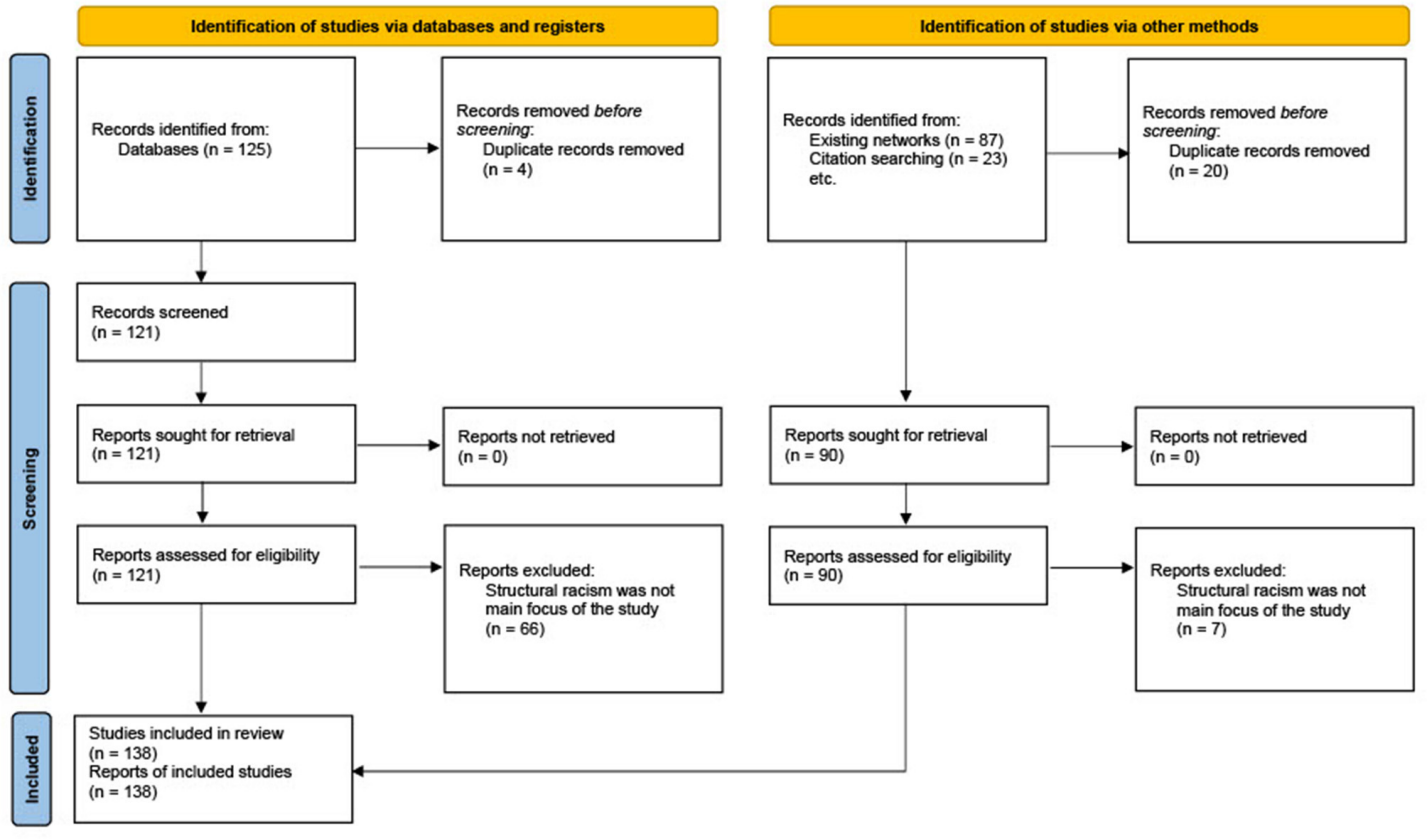
PRISMA flow chart of number of studies included in a scoping review of structural racism with respect to theory, measurement, and methods.

A total of 235 articles were selected (125 from literature review, 87 manually added, and 23 from referenced lists). Duplicate articles (*n* = 24) or articles whose main focus was not structural racism were removed (*n* = 73), leaving a total of 138 articles evaluated (Figure 1). Results were extracted by, and organized into, three broad sections: theory (4–8, 12, 13, 17–24, 37–41, 45, 46, 50, 51, 57, 61, 63, 64, 72, 73, 78, 80, 82–84, 88, 89, 97, 101–103, 106–108, 113, 115), construct measurement (12, 13, 16, 23, 32, 53, 54, 57, 58, 61–63, 65–69, 74, 76, 79, 81, 84–86, 88, 90–92, 98, 106, 109, 110, 112) and study practice and methods (11, 12, 23, 33, 54, 55, 62, 67–70, 74, 75, 77, 82, 87, 91, 93, 94, 104, 109, 111, 115) (Table 2). Table 2 provides an overview of key elements described by the manuscripts for each of the three aforementioned domains. Articles were selected into Table 2 for being illustrative or exemplar with respect to theoretical or methodological relevance, novelty, and scope. Within each domain, several themes are summarized below. The majority of articles discussed were focused on structural racism in the US context. The majority of articles discussed racialized groups in aggregate, focusing on Black (*n* = 125), white (*n* = 105), Hispanic and Latina/o/x/e (*n* = 54), Asian, Asian American, and Pacific Islander (*n* = 34), Native American, Alaska Native, and Indigenous (*n* = 26) and Middle Eastern and North African (*n* = 9) groups, with groups racialized as white often used as comparison group. Few articles included groups racialized as belonging to more than one racial/ethnic group.

### 3.1. Theory

There are several theoretical frameworks conceptualizing structural racism within population health that have been applied to research in epidemiology and related fields. These theoretical frameworks are either informed by or directly incorporate historical context to operationalize structural racism and are inherently political measures. As such, the framing of a research question that includes structural racism needs to acknowledge that these structures are used to maintain white supremacy. Below we provide an overview of conceptualizations of structural racism, arguments for the importance of studying historical exposures, and calls for explicitly discussing both who is harmed and/or benefits from structural racism.

#### 3.1.1. Conceptualization of structural racism in population health (2000-present)

While the phrase “structural racism” has frequently been interchanged with “institutional racism,” these are increasingly considered as two separate concepts (6). Structural racism has been described both in hierarchical terms [i.e., as a subcomponent of systemic racism that operates “above” institutional racism (93, 116)] and as a non-hierarchical, multidimensional concept (73). Gee and Hicken use the analogy of a buckminsterfullerene (a soccer ball-like structure) molecule to describe structural racism as individual institutions bound together by “racialized rules” (i.e., norms, principles, and regulations that govern the behavior of individuals and organizations that reinforce racial hierarchies) (73). A key theme of conceptualizations of structural racism, in contrast with institutional racism, is that structural racism is not solely about institutions acting in isolation.

Structural racism can be both a deliberate action (e.g., restrictive immigration policies that limit or deter immigrant/migrant populations from accessing material resources) (62, 104, 117, 118) as well as “inaction in the face of need” (51) (e.g., lack of workplace protections with respect to occupational COVID-19 exposure) (97). Because structural racism always operates beyond the individual level (93), it can exist without an individual's awareness of it (12). Depending on the particular aspect of interest, structural racism has been both described as visible (22, 53, 67, 104, 119, 120) and invisible (12, 25, 73, 96) to the communities being affected by it. Ultimately, the research question will determine which conceptualizations of structural racism are appropriate to measure, including if and when it is applicable to use participant interviews or questionnaires.

The complex, and at times “invisible” manner through which structural racism shapes population experience demands investigators engage more deeply with theory to conceptualize and articulate exactly how it operates to cause unnecessary illness and death by racialized group. Such theorizing begins by appropriately conceptualizing the meaning of “populations.” As Krieger argues, rather than solely being the “sum of their parts” (i.e., members of a population are defined only by their innate attributes), populations are “bounded complex entities, generated by systemic causal processes” and therefore subjected to the “structured chance” that “drives population distributions of health and entails conceptualizing health and disease” (121). This view of populations makes clear that even the production of meaningful populations is a product of lived social experience. Additional theories frequently employed to interrogate the role of structural racism and population health in research include fundamental cause theory (99), psychosocial theory (122), social production of disease or political economy of health theory (122), Krieger's ecosocial theory (1), and Ford and Airhihenbuwa's Public Health Critical Race praxis (71, 115).

#### 3.1.2. The importance of historical processes on contemporary health outcomes

Structural racism is not a fleeting exposure, but rather a set of systems shaped across time and space. It is common in this research space to evaluate contemporary consequences of historical manifestations of structural racism. Even if structural racism is operationalized as a historical exposure, and therefore cannot be “intervened upon” present-day, the study of historical exposures is important for at least three reasons.

First, studying historical exposures can describe the scale and magnitude of the intervention needed to disrupt “the status quo of health inequity” (123). Rothstein's *Color of Law* (124) provided a rigorous but accessible summary of the motivation for and consequences of historical redlining, or the 1930's state-sponsored discriminatory practice of denying financial services in non-white racial, ethnic, and immigrant group neighborhoods and encouraging loans in white and middle class neighborhoods. Krieger et al. demonstrates that there is a relationship between historical redlining—an exposure that began over 80 years ago—and present-day preterm birth (90). Such long-lasting consequences of a policy or program provides compelling evidence that contemporary efforts to combat the consequences of redlining must address root problems of residential mobility and wealth accrual over the life course and across generations. For example, increasing state allocations of Low-Income Housing Tax Credits and other forms of wealth accrual, such as reparations to address negative socioeconomic status as a result of historical redlining, are needed (17, 90, 125).

Second, historical exposures can help us contextualize and identify important contemporary exposures of structural racism as well as demonstrate how historical structural racism can adapt over time to become contemporary structural racism. For example, studying historical redlining has led to research documenting current day redlining practices using Home Mortgage Disclosure Act data (126). Studies examining the association between contemporary redlining and health using these data have used historical redlining to contextualize this research (55, 58, 106, 127). Although historical redlining was outlawed *via* the Fair Housing Act of 1968, the associations between contemporary redlining and adverse health have demonstrated how even if one aspect of structural racism is addressed (e.g., the Fair Housing Act making the federally-sponsored systematic denial of mortgages based on race or racialized neighborhoods illegal) it does not prevent structural racism from shifting “the work of inequity from one institution to another” (73) (e.g., the systematic denial of mortgages based on race or racialized neighborhoods done by financial institutions).

Third, the study of historical exposures can help us identify interlocking structures and institutions for future study. This can include studies on how systemic racism operates between levels, such as Sewell's study of how discriminatory credit practices between financial institutions (i.e., institutional racism) and government entities (i.e., structural racism) create inequitable dual mortgage markets (106) as well as how different domains within structural racism reinforce each other, such as housing market growth and increased police activity (26–28).

#### 3.1.3. Accountability and shifting away from “actor-less” framing and measures

Because structural racism is used to maintain white supremacy, we cannot simply frame research on structural racism as de-politicized—and therefore actor-less—“social determinants of health” (9, 128). Rather, any epidemiologic research on structural racism requires explicit discussion of who benefits from creating, maintaining, and adapting these structures in addition to who is harmed. As articulated by Krieger (128) regarding the use of the phrase “social determinants of health.”

*Of concern are the health consequences of, say, low income—but not why low income exists. The focus instead is chiefly on…people's relative social standing (a.k.a ‘the ladder'), with little or no consideration of production—that is, who is producing, literally, the goods and services, for whom, for what reason, and at what cost to whom, not only momentarily but also in terms of impact on population and ecologic health*.

*Consequently the arguments and evidence marshaled by these alternative frameworks to reduce or eliminate health inequities by improving social conditions and reducing social inequality typically do not embrace explicit political and economic analysis of whose interests are served by extant inequities. Nor do they call attention to the considerable effort those benefitting from the status quo exert to ensure they continue to accrue their benefits. Profound sociopolitical obstacles to social change are thus unaddressed. Instead, the argument is framed: more equality is better for everyone*.

One example of a shift from an “actor-less” to “actor” approach is Walseman et al.'s study of differential rates of school attendance in the Jim Crow South and cognitive function among Black and white older adults (112). Educational attainment (i.e., an “actor-less” measure that if attributed to anyone is attributed to the individual themselves) has been identified as a key determinant of health with respect to cognitive function, an outcome with persistent Black-white disparities in the US. However, the use of educational attainment alone, measured in years, does not sufficiently capture how structural racism (in the form Jim Crow educational policies) may impact the actual number of years and quality of school attendance, such as *de jure* and *de facto* segregation and school term length. Walseman et al.'s (112) creation of a measure to account for “actors” (i.e., states that legally mandated segregated schooling vs. states that did not) both better described the disparities in cognitive function between Black and white older adults, as well as more clearly articulated which actor(s) are responsible for creating such policies. This can extend to linking other domains of structural racism that precede more proximate social determinants of health. For example, in U.S. Southern states, counties with a higher concentration of slaves in 1860 experienced a slower decline in heart disease mortality among present-day Black populations compared to white populations (32).

### 3.2. Measurement

Work on operationalizing measures of structural racism has progressed on several fronts. First we discuss literature on operationalizing “race” and structural racism, as well as data sources used to operationalize these two constructs. We then describe uni- and multi-dimensional measures of structural racism, and conclude with special considerations for incorporating different levels of systemic racism and intersectionality in structural racism and health studies.

#### 3.2.1. Operationalizing “race”

As with any study, special attention needs to be paid to how study constructs are operationalized into valid and reliable measures. Adkins-Jackson et al. enumerates the challenges of operationalizing structural racism in epidemiological research (12). Because the health consequences of structural racism are typically racially differential, it is important to start with clarity about how “race” is conceived, measured, and operationalized in causal models and analyses. In the edited collection, *White Logic, White Methods* (2008), chapters written by James, Marks, and Khalfani et al. are instructive with respect to the use of “race” as a variable in research (129). Considerations for operationalizing race and ethnicity should extend beyond using National Institute of Health categories (130), and should include whether the research question concerns racial and/or ethnic self-identification, how individuals are racialized, and whether or not these classifications obscure racial and ethnic populations *via* data aggregation (12, 93, 131).

#### 3.2.2. Operationalizing structural racism

The operationalization of structural racism often involves the use of secondary data sources, which may or may not accurately represent the construct that is being operationalized. One example is how police-recorded crime data is operationalized by Lodge et al. (94, 95), which utilizes police-reported crime not solely as a measure of criminal activity, but rather as combination of criminal offenses and police activity, including police violence and surveillance. Often police-reported crime data is used to operationalize the construct of “neighborhood crime” to assess the relationship between neighborhood-level violence and health. However, the use of police-reported data requires the assumption that police reported crime is both an accurate measure of true neighborhood crime, and possibly the additional assumption that police behavior does not affect health, despite that policing—particularly racialized policing—is a form of violence (23, 132). Special care should be taken to find an agreement between construct validity and the data used to operationalize these constructs.

#### 3.2.3. Data source considerations

Data sources commonly used to operationalize structural racism include US Census products, such as the US Decennial Census and the American Community Survey (ACS). However, their limitations should also be taken into consideration. In addition to using appropriate margins of error and paying heed to coverage error (133–137), it is important to recognize that variables from the Census, without proper framing, obscure structural processes and actors, which as Riley notes, “makes them insipid proxies for structural racism” (101). While ACS data contains a broad range of data on material resources, it may implicitly or explicitly allow researchers to systematically ignore structural processes that are harder to measure. Riley provides the example of scholars attributing racial inequities to proxies of structural racism, such as neighborhood poverty, as opposed to the processes of structural racism, such as discriminatory housing markets and the systematic underfunding of majority-Black schools (101).

Other potential data sources to operationalize structural racism include but are not limited to mortgage reporting data (126) and less commonly the Freedom of Information Act (FOIA) (138, 139). However, obtaining FOIA data may be difficult and time-consuming to procure. Researchers can partner with or utilize already completed FOIA requests from legal organizations, such as the American Civil Liberties Union's FOIA Collection (140).

#### 3.2.4. Unidimensional measures of structural racism

As we have noted previously, structural racism is a complex process involving multiple institutions and processes over time and space. Unidimensional measures include single measures, policies, and indicators, which provide greater ease of use and interpretation (86). The tradeoff is that any single measure is by definition incomplete and less representative of the full effect of structural racism. To capture the entirety of structural racism in a single measure is likely impossible. We echo Jahn that the “best” measure(s) of structural racism are study- and question-dependent (86). Adkins-Jackon et al. (12), the invited commentary by Jahn (86), and Hardeman et al. (13) are all foundational texts for the study of structural racism and health, and they provide an excellent overview of commonly used unidimensional and multidimensional measures of structural racism. As noted by Jahn, operationalizing a specific structural process (e.g., policy) by using a unidimensional measure helps focus on specific policy or program action, but are not without their limitations (86).

Hardeman et al. (13) extends Phelan and Link's fundamental cause theory (99), elucidating that while studies that focus on singular dimensions of structural racism may help develop precise policy, they (1) may not anticipate how other forms of structural racism “accommodate” an intervention to maintain the status quo and (2) do not fully capture the ramifications of a given policy. For example, while the use of stay-at-home orders may prevent COVID-19 transmission and possibly reduce racial and ethnic inequities in COVID-19 transmission, the use of police and racialized enforcement of public health ordinances are antithetical to these efforts (87).

#### 3.2.5. Multidimensional measures of structural racism

A growing body of scholarship operationalizes structural racism as an explicitly multidimensional construct and measure (12, 13, 68, 69, 73, 74, 84, 86, 91, 92, 98, 106). Multidimensional measures can be created with latent-class models incorporating indicators across domains of structural racism as illustrated by Chantarat et al. (68). Multidimensional measures can also be used as an approach to characterize effects within and between different levels of systemic racism as illustrated by Sewell (106), and as an approach to intersectionality as illustrated by Homan et al. (84).

Broadly, latent constructs (i.e., phenomena that are real but not directly observable, like structural racism) can be measured by a variety of statistical techniques, including principal component analysis for continuous variables and latent-class analysis for categorical variables. Strengths of utilizing this method include the ability to incorporate multiple domains of structural racism and the ability to address issues of multicollinearity (12, 13, 68, 86). Using multiple unidimensional indicators of structural racism in regression analyses are likely to be highly correlated with each other. With latent class models the assumption of shared variance is both theoretically appropriate and statistically addressed. While the main limitation for using latent constructs to create multidimensional measures of structural racism is the general assumption that the shared variance between the inputs for the latent-class measure represents construct validity (i.e., the variance between the measures does not represent anything other than structural racism) (12), this method may not be appropriate depending on the study question. First, as highlighted by Jahn, findings from studies that operationalize structural racism as a latent class measure may be less translatable into specific, single, discrete interventions (86). Second, the development of latent-classes may be historically and spatially contingent: while this method is ideal to measure structural racism both in magnitude and changes by domain over time in a given place, as noted in Chantarat et al. (68), it is unclear if this method is ideal for comparing two places at the same time. Future studies to compare latent constructs of structural racism from one place vs. another could be useful as a means for characterizing structural racism even in the absence of a health outcome.

#### 3.2.6. Measuring structural racism with other levels of systemic racism

Structural racism can also be operationalized in a way that measures its relationship to other levels of systemic racism. Sewell used neighborhood credit refusals, racialized credit refusals, neighborhood private credit use, and racialized private credit use to develop a four-dimensional approach to describe the dual mortgage market (106). Sewell developed this approach to capture the effects that the relationship between institutional (i.e., credit refusal within financial institutions) and structural (i.e., interactions between financial institutions and the government) racism within a given domain (neighborhood) have on health (106). One key strength of this approach is that it methodologically “maps” well to Gee and Hicken's definition of structural racism, where institutions are connected *via* “racialized rules” that binds individual institutions together to enforce structural racism (73).

#### 3.2.7. Incorporating intersectionality

Because systems of oppression are “interlocking, mutually constituted, and reinforcing,” (84) incorporating intersectionality can be of interest when studying the effects of structural racism (13, 29, 69, 84). Introduced by legal scholar Kimberlé Crenshaw, intersectionality is used as both a framework and analytic tool to understand overlapping systems of oppression, such as racism, sexism, classism, and other forms of inequity (29). “Structural intersectionality” developed by Homan et al. is an approach to incorporate intersectionality into quantitative research by measuring these systems at the contextual level as opposed to the individual level (84). In their study, structural intersectionality was operationalized as three systems of oppression measured at the state level (structural racism, structural sexism, and structural classism) to evaluate the prediction of self-rated health. Structural racism was operationalized as an index consisting of nine different state-level indicators across five domains, structural sexism was operationalized as an index consisting of six different state-level indicators across four different domains, and structural classism was operationalized by calculating the state-level Gini coefficient for income inequality (30, 84). Strengths of this approach include a novel way to measure intersectionality at the structural level, as well as the potential to more accurately reflect the multidimensionality of structural racism across several domains (84, 86). Limitations include the assumption that all indicator variables being summarized have equal weight to the measure, concerns over sample size, and challenges with the selection of which variables to include to represent structural intersectionality (84, 86). However, utilizing theory pertaining to these topics (structural racism, structural sexism, intersectionality) along with a discussion of limitations for variables selected and not selected can help communicate the theoretical and methodological assumptions used by the researchers (12, 84).

### 3.3. Practice and methods

There are existing practices and methods that have been applied to structural racism and health studies. Both Adkins-Jackson et al. (12) and Hardeman et al. (13) cover many of these existing practices in-depth, as well as specific considerations when applying these practices for structural racism and health studies. We reiterate the recommendation by both studies to consult or partner with subject matter experts, colleagues from outside of epidemiology, and/or affected communities, all of whom should be compensated for their expertise and labor. Below we expand on current practices and methods, discuss advances and limitations for methods specifically developed to assess the impact of structural racism, and briefly discuss challenges in structural racism and health studies with respect to causal inference.

#### 3.3.1. Defining a meaningful study population and comparison group

Conceptualizing a meaningful definition of a study population is important for studying structural racism. While historically the default approach is to use white populations as the reference group, depending on the research question this may not be appropriate (11). For example, an investigator may want to understand intracategorical intersectionality (i.e., the intersections of experiences within those of a marginalized group identity), such as how risk varies by gender by racial and ethnic categories, or class variation within race. Revisiting Homan et al. the authors examine above vs. lower than average exposure to structural racism, sexism, and income inequality within four different populations (Black Women, Black men, white women, and white men), as opposed to examining differences between these populations, as the systems of oppression do and do not “favor” these populations in different ways (84). Identifying an appropriate comparison group also applies to conducting within-group analyses. In addition to disaggregating conventionally used racial and ethnic categories discussed earlier, within-group analyses can reveal both new manifestations and differing experiences of structural racism (45, 71, 78). Selecting the ideal population and comparison group emphasizes the need for theory to articulate who is harmed, or, if applicable, benefits, from structural racism.

#### 3.3.2. Qualitative study design to develop domains of structural racism

Given the complexity of measuring structural racism, some empirical (quantitative) studies may not be appropriate, and qualitative or mixed-method studies should be considered (12, 13). For example, qualitative study design (e.g., focus groups) may be ideal for research questions identifying domains of structural racism relevant to their outcome or population, as stakeholders may be more intimately familiar with these interlocking institutions (67, 91, 104, 109). Examples include Sabo et al.'s (104) use of mixed methods to illustrate how anti-immigrant policies may lead to institutional practices that lead to race and ethnicity being conflated with immigration status among residents of Mexican descent, Chambers' (67) work conducting focus groups with Black women across the reproductive lifespan to generate and validate domains of structural racism, and LaFave et al.'s (91) use of interviews and focus groups with older Black adults, discrimination researchers, and stakeholders to design a measure of “structural racial discrimination” for older Black Americans (74, 88).

#### 3.3.3. Study designs for quantifying historical and contemporary policy changes over time

A number of methods and statistical techniques have been applied to structural racism and health studies. Many of these designs and techniques overlap with each other and should not be considered as mutually exclusive. Age-period-cohort (APC) designs utilize historical population changes in health to discern if health effects are driven by processes of biosocial aging of individuals, period-specific shocks that affected all ages groups, or cohort-specific manifestations of structural racism experienced by generational birth cohorts, such as examining changes in Black and white premature mortality rates following the abolition of Jim Crow *via* the 1964 Civil Rights Act (33). One strength of APC is the potential to examine distinct scales of time-varying structural racism as an epidemiologic interaction, as done in an APC study on obesity prevalence in the US (141). For example, one study could assess the effect of the GI Bill on men who would have been eligible by a given health outcome, while also examining the difference in those outcomes by race over time. Challenges for APC include the use of APC designs as an exploratory descriptive tool (i.e., not appropriate for causal inference), and issues of causal non-identifiability (142). Regression discontinuity, interrupted time series, and difference-in-difference study designs are ideal for structural racism where there is either a time-varying or abrupt change in a policy or structural process. Examples include racial and ethnic disparities in access to care following Medicare eligibility for regression discontinuity (111), racial disparities in arrests following COVID-19 stay-at-home orders in 4 cities for interrupted time series (87), and the association of the 2012 Deferred Action for Childhood Arrivals (DACA) program on birth outcomes among children of Mexican-immigrant birthing parents (77). These study designs are ideal to measure dramatic inter-state (or other unit of comparison) variations, as state-level variations are an important unit of analysis for structural racism in the US (13).

However, with these aforementioned study designs, it may be difficult to isolate the change of one policy, as states (or other units of comparison) are unlikely to vary on just the one change of interest. Adkins-Jackson et al. (12) discusses legal scholar john a. powell's (31) scholarship on structural racism and states that measuring changes at one point in time does not adequately capture structural racism's continuous impact on health. Additionally, Hill (31) critiques the focus on discrete changes; by using historical redlining as an example, Hill argues that “while redlining is important to understand in the historiography of racism, this ongoing search for legacy impacts of redlining overdetermines contemporary outcomes of past practices while missing the suite of policies and practices that informed spatial racism at that time and today” such as restrictive racial housing covenants, “bluelining” (the use of racial classifications of neighborhoods by retailers) and “greenlining” (the targeting of Black and other non-white homeowners for subprime mortgages) (31).

#### 3.3.4. Study designs incorporating place and geospatial methods

Because structural racism affects where groups live, attend school, work, and play, incorporating “place” can be an informative approach to structural racism and health studies. Such studies can be broadly categorized as place-based analyses (analyses that incorporate units of geography) and spatial analyses (studies that incorporate geospatial contiguity and proximity into analyses). Examples of place-based analyses include (1) the association of the index of concentration at the extremes for preterm birth and infant mortality for Black women in California, (2) examining the joint effects of state-level indicators and income inequality on small-for-gestational-age births, and (3) the development of new measures of structural racism, such as the “racial opportunity gap”(66, 110, 143). Examples of geospatial methods include (1) examining the legacy of slavery in county-specific temporal and geographic declining patterns in heart disease mortality and (2) measuring the association between living in a high-police contact neighborhood and preterm birth in Minneapolis (32, 81). While a strength of these analyses is that manifestations of structural racism are most readily recognizable through place-based effects, there are methodological and theoretical challenges. A key methodological challenge is selecting an appropriate unit of geography for studies on structural racism and health, as detailed in Hardeman et al. (13). A key theoretical challenge is that place-based analyses in and of themselves do not challenge normative framing by which racial/ethnic disparities are produced and maintained, and without this framing can be used to reinforce racist assumptions about communities and health (101). All of these designs highlight the importance of interdisciplinary scholars and/or community partners for the purposes of study conceptualization, measurement, and interpretation of results.

#### 3.3.5. Incorporating complexity through complex systems modeling

Methods have been minimally developed to estimate the impact of the multidimensional nature of structural racism relative to unidimensional measures of structural racism. There have been calls to adopt tools for the study of complex systems, including agent-based modeling (ABM), as a means to investigate structural racism and population health (12, 93, 144). ABMs are simulations of complex systems with a key advantage of distilling the salient factors of a system while still permitting complex, non-linear patterns to emerge by designating agents (people, institutions), designating environmental states (structures), and setting rules by which agents interact with each other and the environment (145, 146). ABMs have been identified as a potential tool to try “*in silica*” tests of interventions as one step in developing real-world interventions, and have been developed to simulate counterfactual outcomes in epidemiology (144–148). ABMs can help address (1) the lack of an appropriate counterfactual in observational data given that structural racism affects every aspect of marginalized groups' lives beginning *in utero* and (2) the effects of structural racism that are dependent and closely correlated with one another (149–152). ABMs also address a common limitation in epidemiologic studies evaluating policy interventions for structural racism: the possible unintended consequences and sequelae of interventions either at the individual, institutional, or structural level (13, 145, 148). Examples of ABMs include, (1) exploring the role of economic segregation on creating differences in income and subsequent healthy eating behaviors, (2) simulating two interventions on city-level violence, and (3) estimating the impacts of a free bus policy on depression among older adults (147, 153, 154). While ABMs are a promising tool, there are a number of challenges, including but not limited to the use of strong assumptions about individuals and systems, model calibration, and model validation (155). The parametric g-formula has been proposed as an “in-between” method between traditional epidemiologic methods and ABM, and has been used in structural racism and health studies (74, 156).

#### 3.3.6. Causal thinking and quantitative causal inference

It should be stressed that quantitative causal inference is distinct from solely identifying causes or “proving causality,” but rather used to empirically “help us predict what would happen under different interventions, which requires our commitment to define the interventions of interest” (157). Special care should be taken to frame quantitative causal inference studies not as proving or disproving structural racism as a cause of poor health or health inequities, but rather, as Graetz et al. point out, “the starting point” in developing a research question (74).

There has been active discourse surrounding social epidemiology and quantitative causal inference—specifically the potential outcomes framework—that extends to studies on structural racism and health (149, 150, 152). While causal inference methods have not been as well-developed for social epidemiology, a branch of structural racism and health research could benefit under social epidemiology's contribution to the potential outcomes framework through the framework's approach to developing a more rigorous and transparent effect of interest, such a clarifying between “race,” “racialization,” and “racism” as exposures in research (6, 150). Considerations for social exposures that can be extended to structural racism and quantitative causal inference include exchangeability (158, 159), structural positivity (i.e., structural confounding) (160), the potential conservativeness of consistency (152, 161), and stable unit treatment value assumption (SUTVA) violations (152). Mediation analysis methods have been specifically developed to address differences in health by social mediators, including one study, Graetz et al. on time-varying structural racism with respect to cardiometabolic risk over the lifecourse (74, 162).

## 4. Discussion

In this scoping review we organized and summarized articles on structural racism and health with respect to theory, measurement, and methods, and provided a few in-depth descriptions of relevant literature for each topic. This review builds upon topics discussed by Adkins-Jackson et al. (12) and Hardeman et al. (13) by providing a more in-depth discussion of framing and measures that address accountability in structural racism studies, measuring structural racism with other levels of systemic racism, and incorporating complex systems study design. This review also introduces a summary of relevant literature on causal inference and structural racism. Below we offer an assessment of recommendations implicitly or explicitly proposed by articles read for this scoping review, divided by theory, measurement, and practice and methods. Scholars new to this literature and seeking resources for research best practices should supplement our summary below with the reviews by Adkins-Jackson et al. (12) and Hardeman et al. (13).

### 4.1. Best practices and recommendations: Theory

- Hire team members or consultants with expertise in studying structural racism. This includes both academics and community members, all of whom should be compensated for their work (12, 13, 93).
- Review literature—particularly theory and methods—outside of epidemiology, including history, sociology, anthropology, geography, etc. (12).
- Conceptualize and articulate the population of interest, not just as defined by their individual attributes, but as shaped by their shared social and relational experience that may be both historically and spatially contingent (121).
- When applicable, be explicit about how a given operationalization of structural racism is cognizant of the actors involved, and who is harmed and who benefits from the process being proxied by the measure. Relatedly, if the harms are due to the restriction of “limited” material resources (e.g., wages, housing), it may be helpful to explore why those resources are allocated in racialized ways, and which actors or forces are resistant to changes (128).
- Use a historical and theoretical approach, as there can be no discussion of structural racism without a discussion of the potential pathways through which structural racism produces disease. Given that structural racism is a system, “making sense” of structural racism requires a framework. Taking an ahistorical or atheoretical approach ignores the processes that create and maintain structural racism, and therefore impedes meaningful public health action (9, 12, 13, 128).
- Critically appraise inclusion of variables pertaining to race in statistical models (12). Utilizing theoretical frameworks combined with directed acyclic graphs (DAGs) can help prevent inadvertent adjustment for factors that are in fact consequences of structural racism and thus mediating rather than confounding associations with health (93, 163).

### 4.2. Best practices and recommendations: Measurement

- Become familiar with current measures of structural racism, their theoretical underpinnings, and evaluate which measure(s) are most appropriate to use given your research question. Adkins-Jackson et al. (12), Hardeman et al. (13), and Jahn (86) are foundational texts for the measurement of structural racism and health.
- Critically evaluate the motivations, “baked-in” assumptions, and limitations of secondary data sources (89, 94, 101, 129).
- Assess the scale at which a measure was developed for vs. the scale at which the process occurs (e.g., individual, community, societal, etc.) (13). For example, measuring census tract differences in educational attainment may be of relevant interest, but if a study is to identify areas of intervention, then the appropriate unit of analysis may be at the school district level or above (7, 13).
- Recognize that any approach is ultimately a data reduction method in which we are trying to simplify the complex ways structural racism operates. Research operationalizing structural racism should discuss relevant limitations (12).

### 4.3. Best practices and recommendations: Practice and methods

- Consult or partner with subject matter experts, colleagues from outside of epidemiology, and/or affected communities, all of whom should be compensated for their expertise and labor (12, 13, 67, 93).
- Utilize qualitative and mixed methods, particularly with respect to defining or validating domains of structural racism (12, 13, 67, 91, 104).
- Do not assume that white populations are the ideal reference population for studies on structural racism (11, 71, 78).
- Utilize methods that highlight structural racism's continuous, pervasive, nature, as opposed to defining structural racism as discrete, individual events (12, 31, 74).
- Recognize that quantitative causal inference is distinct from identifying various forms of structural racism as causes of poor health. These frameworks and methods are one of many ways to empirically assess the impact of structural racism on health and the effect of potential interventions (74, 150).

### 4.4. Limitations

This scoping review has the following limitations. The first limitation is that articles were restricted to those written in English, with the overwhelming majority of articles focusing on structural racism in the US. While this resulted in no exclusion of articles from the scoping review, we recognize that this both limits our understanding of structural racism and relevant data sources to studies being conducted in the US. Second, due to our search strategy, not all articles generated by Google Scholar were evaluated for inclusion into the scoping review (10). Because of this, relevant articles that were ranked lower by Google Scholar were missed based on our exclusion criteria (59). For example, one article on a conceptual model and public health recommendations for structural racism against Asians (164) and another article on the interlocking impacts of colonialism and racism on Filipinx/a/o Americans (165) were both missed by this search strategy, despite meeting search criteria.

### 4.5. Conclusion

The purpose of this scoping review is to summarize current literature on structural racism with respect to theory, measurement, and methods commonly used and highlight key works in this field. In doing this, we hope to provide a starting point for trainees and public health researchers who are not already deeply grounded in this work. While recent energy and renewed interest with respect to this topic can provide a new lens through which we can understand and ultimately intervene upon the reproduction of racial and ethnic health inequities, in the spirit of minimizing future potential harms inflicted on marginalized communities, investigators have a duty to use the term “structural racism” with critical precision, grounded in the works and definitions widely established by scholars outside of the traditional frameworks of public health (11, 100).

## Data availability statement

The original contributions presented in the study are included in the article/supplementary material, further inquiries can be directed to the corresponding author.

## Author contributions

SW, AM, and MK designed the research, conducted study procedures, analyzed the data, provided critical input to the manuscript draft, and approved the final manuscript draft. SW wrote the initial draft of the manuscript. All authors contributed to the article and approved the submitted version.
